# La gigantomastie: à propos d'un cas et revue de la littérature

**DOI:** 10.11604/pamj.2014.18.154.2749

**Published:** 2014-06-18

**Authors:** Nisrine Mamouni, Sanaa Erraghay, Aya Oufkir, Hanane Saadi, Chahrazed Bouchikhi, Abdelaziz Banani

**Affiliations:** 1 Service de Gynécologie-Obstétrique I, CHU Hassan II, Fès, Maroc; 2 Service de Chirurgie Plastique, CHU Hassan II, Fès, Maroc

**Keywords:** Glande mammaire, hypertrophie, puberté, traitement, pronostic, mammary gland, hypertrophy, puberty, treatment, prognosis

## Abstract

L'hypertrophie virginale ou gigantomastie est une mastopathie rare de cause inconnue. Elle survient au moment de la puberté et se manifeste cliniquement par un accroissement rapide et bilatéral du volume des seins. Les dosages hormonaux sont habituellement normaux et la biopsie du sein montre une accentuation du tissu mésenchymateux. Du fait des complications mécaniques et psychologiques liées aux poids et volume excessifs des seins, un traitement chirurgical rapide et efficace s'impose. Le But est de rapporter un cas rare de gigantomastie juvénile, discuter les éventualités thérapeutiques ainsi que le pronostic.

## Introduction

La gigantomastie est une affection bénigne rare qui touche habituellement la femme jeune à la période pubertaire dont l’étiologie inconnue pour laquelle on évoque une hypersensibilité aux hormones sexuelles. Du fait des complications mécaniques et psychologiques liées aux poids et volume excessifs des seins, un traitement chirurgical rapide et efficace s'impose. Le but est de rapporter un cas rare de gigantomastie juvénile, discuter les éventualités thérapeutiques ainsi que le pronostic.

## Patient et observation

**Figure 1:**
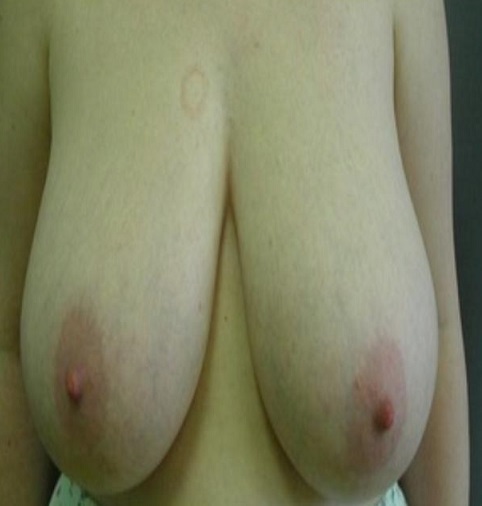
Photographie des seins en préopératoire (position demi assise)

**Figure 2:**
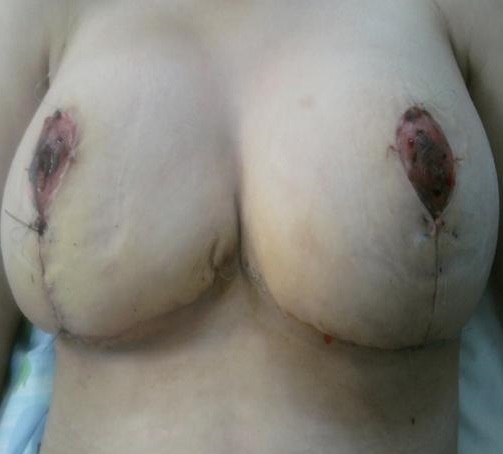
Photographie en post opératoire, Jour 10

Il s'agit de mademoiselle S.T, âgée de 15 ans, menarchée à l’âge de 12 ans, qui présente depuis 3 ans une augmentation progressive du volume des seins sans écoulement mamelonnaire associé. L'examen clinique objective une augmentation asymétrique de la taille des deux seins; le sein droit plus développé que le sein controlatéral avec présence d'une hypœsthésie de la plaque areolomammelonnaire Figure 1. L’échographie mammaire, difficile à réaliser, ne montre pas de nodule intra mammaire kystique ou tissulaire. Le dosage hormonal d’œstrogène et de Prolactine est normal. La patiente a bénéficié d'une réduction mammaire (technique en T inversé). L'intervention est pratiquée sous anesthésie générale, en position demi-assise, bras le long du corps. Elle dure environ 3 heures. Un drainage par drain de Redon ainsi qu'un pansement compressif sont mis à la fin de l'intervention Figure 2. La durée de l'hospitalisation a été de 4 jours. Un soutien-gorge de sport en tissu élastique de maintien a été prescrit, à porter jour et nuit pendant 6 semaines après l'intervention (soutien-gorge sans armature, s'ouvrant si possible par devant). L’évolution après un recul de 24 mois est favorable.

## Discussion

La gigantomastie est une forme exubérante d'hypertrophie mammaire définie par des seins dont le volume mammaire dépasse 1500 cm3, les normes variant habituellement de 200 à 350 cm^3^. [1] La gigantomastie est uni ou bilatérale, d'installation brutale et d’évolution rapide. Le sein est volumineux, ferme, indolore, d'aspect inflammatoire, siège d'une macération cutanée au niveau du sillon sous-mammaire, responsable de véritables lésions infectieuses voire même de nécrose.

Cette hypertrophie mammaire est à l'origine d'une véritable détresse psychologique et d'un grand malaise physique. Elle occasionne souvent des modifications posturales du fait de l'excès de poids vers l'avant que la patiente adopte, plus ou moins consciemment, dans le but de dissimuler la taille de ses seins avec une position de cyphose dorsale, épaules projetées en avant. Ces attitudes conditionnées peuvent persister même après l'intervention.

Les formes habituelles de cette entité exceptionnelle sont: La forme juvénile qui touche la fille en période pubertaire dont La physiopathologie est très discutée. Le facteur le plus important serait dû à un déséquilibre hormonal. L'augmentation du nombre des récepteurs d'estrogène et/ou de progestérone a été mise en cause. Noczinska et al. [2] ont retrouvé dans leur étude un taux élevé de récepteurs aux estrogènes, alors que cette hypothèse n'a pas été retrouvée par Lafrenière et al. [3]; La forme gravidique consécutive à une évolution monstrueuse de l'hyperplasie épithéliale habituelle survenant au cours ou au décours de la grossesse; La gigantomastie iatrogène secondaire à une prise médicamenteuse.

En 1973, Desai [4] rapportait le premier cas de gigantomastie compliquant la prise de D-pénicillamine chez une patiente atteinte d'une polyarthrite rhumatoïde, et depuis des cas similaires ont été décrits [5]. D'autres médicaments ont été incriminés à savoir le Néothétazone, la cyclosporine et la bucillamine [6, 7].

La gigantomastie idiopathique est encore plus exceptionnelle, atteignant la femme adulte de plus de 20 ans en dehors de la grossesse [8]. Histologiquement, on observe une hypertrophie du tissu conjonctif plutôt qu'une hyperplasie du tissu glandulaire. Hedberg et al. [9] rapportent une accumulation intracellulaire d'une substance plutôt qu'une prolifération du tissu conjonctif.

L'imagerie n'occupe pas une place importante dans le diagnostic car les seins ont un aspect très dense. Il n'y a pas de conduite thérapeutique standard de la gigantomastie vu le faible nombre des cas rapportés dans la littérature. La majorité des auteurs proposent l'hormonothérapie comme traitement de première intention en cas de gigantomastie gravidique par médroxyprogestérone, dydrogestérone, tamoxifène, danazol, les androgènes ou la gosereline [4–8]. À fortes doses, ce médicament peut entraîner l'arrêt voire même la régression de la gigantomastie surtout dans sa forme gravidique [9]. Le Tamoxifène serait utile pour prévenir les récidives après mammoplastie de réduction [10]. Les corticoïdes et les diurétiques, peuvent être utilisés prudemment [10]. En cas de non-réponse au traitement médical, la chirurgie s'impose avant l'installation de la nécrose.

Les impératifs de la chirurgie de réduction mammaire sont: la diminution du volume mammaire, le repositionnement et la viabilité de la plaque aréolo-mamelonnaire, les cicatrices cutanées les plus discrètes possibles et une forme harmonieuse et stable, adaptée à la morphologie de la patiente et à ses désirs. La plastie mammaire de réduction (PMR) est désormais une intervention chirurgicale courante en cas de gigantomastie juvénile, motivée par des raisons essentiellement fonctionnelles, psychologiques et/ou esthétiques. Il s'agit d'une chirurgie réalisée le plus souvent à partir de l’âge de 15-16 ans pour que les seins terminent leur maturation [4, 5] et les qualités mécaniques de la peau sont meilleures, tout en tenant compte du caractère fonctionnel des seins (allaitement). Peu d'articles ont été publiés tant sur la prise en charge de l'hypertrophie mammaire juvénile que sur les possibilités d'allaiter après une PMR [9, 11, 12]. Les techniques les plus souvent utilisées sont le Mc Kissock, la réduction à la Thorek, ou le T inversé comme le cas pour notre patiente.

Après mammoplastie l’évolution est marquée par la fréquence des récidives dans un délai de quelques mois, voire des années. La grossesse en serait une des causes, en raison de l'hyperœstrogénie associée [11–15]. Dans le cas où une intervention est décidée, il faut respecter un délai de deux ans avant d'envisager une grossesse.

Des mammographies bilatérales, et/ou échographie mammaire, sont nécessaires chez les femmes de plus de 35 ans, ou chez celles présentant des antécédents personnels et/ou familiaux particuliers. Dans tous les cas, il est recommandé de faire pratiquer une mammographie quelques mois après l'intervention, afin de servir de référence à la surveillance ultérieure des seins.

## Conclusion

La gigantomastie est une entité rare, d’étiologie indéterminée. Elle pose le problème du traitement chirurgical qui peut être très rarement radical de nécessité. Un suivi à long terme est nécessaire, des récidives étant possibles, conduisant dans les cas extrêmes à une mastectomie bilatérale avec reconstruction mammaire immédiate ou secondaire.
